# Targeting autoreactive germinal centers to curb autoimmunity

**DOI:** 10.18632/oncotarget.21701

**Published:** 2017-10-09

**Authors:** Søren E. Degn, Cees E. van der Poel, Michael C. Carroll

**Affiliations:** Søren E. Degn: Department of Biomedicine, Aarhus University, 8000 Aarhus C, Denmark; Program in Cellular and Molecular Medicine, Boston Children’s Hospital, Boston, MA, USA

**Keywords:** autoimmunity, B cells, systemic lupus erythematosus, germinal genters, lymphocytes

Autoimmune diseases have been on the rise in the past decades, particularly in Western societies [1]. Today, autoimmune disorders rank third on the list of most prevalent causes of morbidity and mortality in the Western world. Many autoimmune conditions, such as systemic lupus erythematosus (SLE) and rheumatoid arthritis (RA), are characterized by the presence of affinity-matured, class-switched antibodies to autoantigens [2]. Such autoantibodies are thought to drive pathogenesis, and are generated in specialized microanatomical structures in secondary lymphoid tissues, which are termed germinal centers (GCs) [3].

In GCs, B cells cycle between the dark zone and the light zone. In the dark zone, B cell clones proliferate and undergo somatic hypermutation (SHM), leading to introduction of spontaneous mutations in their immunoglobulin V(D)J loci. In the light zone, mutant B cells scan follicular dendritic cells (FDCs) for antigen. B cells that are able to take up antigen through their B cell receptor (BCR) can present antigen-derived peptides to cognate T follicular helper (Tfh) cells. Tfh cells then stimulate these B cells to return to the dark zone for another round of division and hypermutation. In this iterative Darwinistic process of diversity generation and selection based on affinity, B cell clones which recognize antigen with increasing affinity are selected and expanded, and give rise to plasma cells producing high-affinity antibodies.

For this reason, therapeutic strategies to block autoimmune GC reactions have been explored clinically, most notably through blockade of the B-T cell interaction through blocking anti-CD40L (CD154) antibody [4]. However, although efficient across a range of murine models, and tentatively efficacious in humans, clinical trials were thwarted by off-target effects of anti-CD40L in platelets, leading to thromboembolism [5].

We recently investigated the clonal evolution of autoreactive GCs in the murine 564Igi model, a knock-in of the heavy and light chains of an autoantibody specific for ribonuclear complexes [6]. Surprisingly, although knock-in B cells dominate the circulating repertoire, the majority of B cells participating in the GC response did not carry the receptor specified by the knock-in. The same was also true when mixed bone marrow chimeras were generated, in which the 564Igi knock-in cells were genetically segregated from the wild-type repertoire; i.e., spontaneous autoreactive GCs emerged, and were predominantly composed of wild-type B cells. Thus, a single, hard-wired autoreactive B cell clone was able to initiate systemic autoimmunity and drive formation of GCs, which recruited presumed protoautoreactive B cells from the endogenous repertoire. These GCs became self-sufficient and persisted even after ablation of the initiating 564Igi cells. Autoreactive wild-type cells furthermore evolved and affinity-matured in a manner comparable to GC responses to foreign immunogens, and this evolution was mirrored serologically by functional epitope spreading, leading to autoantibody deposition in the kidneys [6].

The chronic and progressive nature of epitope spreading observed clinically calls for early interventions, even before disease onset. Our recent findings underscore the central role of GCs, at the heart of the autoimmune response, and call for a rejuvenated effort to develop strategies that block this process. One approach may be to engineer CD40L blocking strategies that circumvent the off-target effects of anti-CD40L antibody therapy, which appear to have been driven largely by Fc-interactions [7]. However, being broadly expressed on activated CD4 T cells, CD40L may have important auxiliary roles beyond supporting GC responses, for example in Th1-mediated immunity, through interactions with CD40 expressed on monocytes and dendritic cells. Accordingly, alternative strategies could instead target GC B cells directly, or via other indirect routes. One option might be to influence the cytokine environment that fuels autoreactive GC propagation, as suggested in another recent study from our group where we found that blocking interferon alpha signals by FDCs ameliorated autoimmunity [8]. Notwithstanding such considerations of specific molecular and cellular targets, seeing as GCs are the ‘furnace of autoimmunity’, a deeper understanding of autoreactive GC dynamics and alternate approaches to target autoreactive GCs are urgently needed.

**Figure 1:**
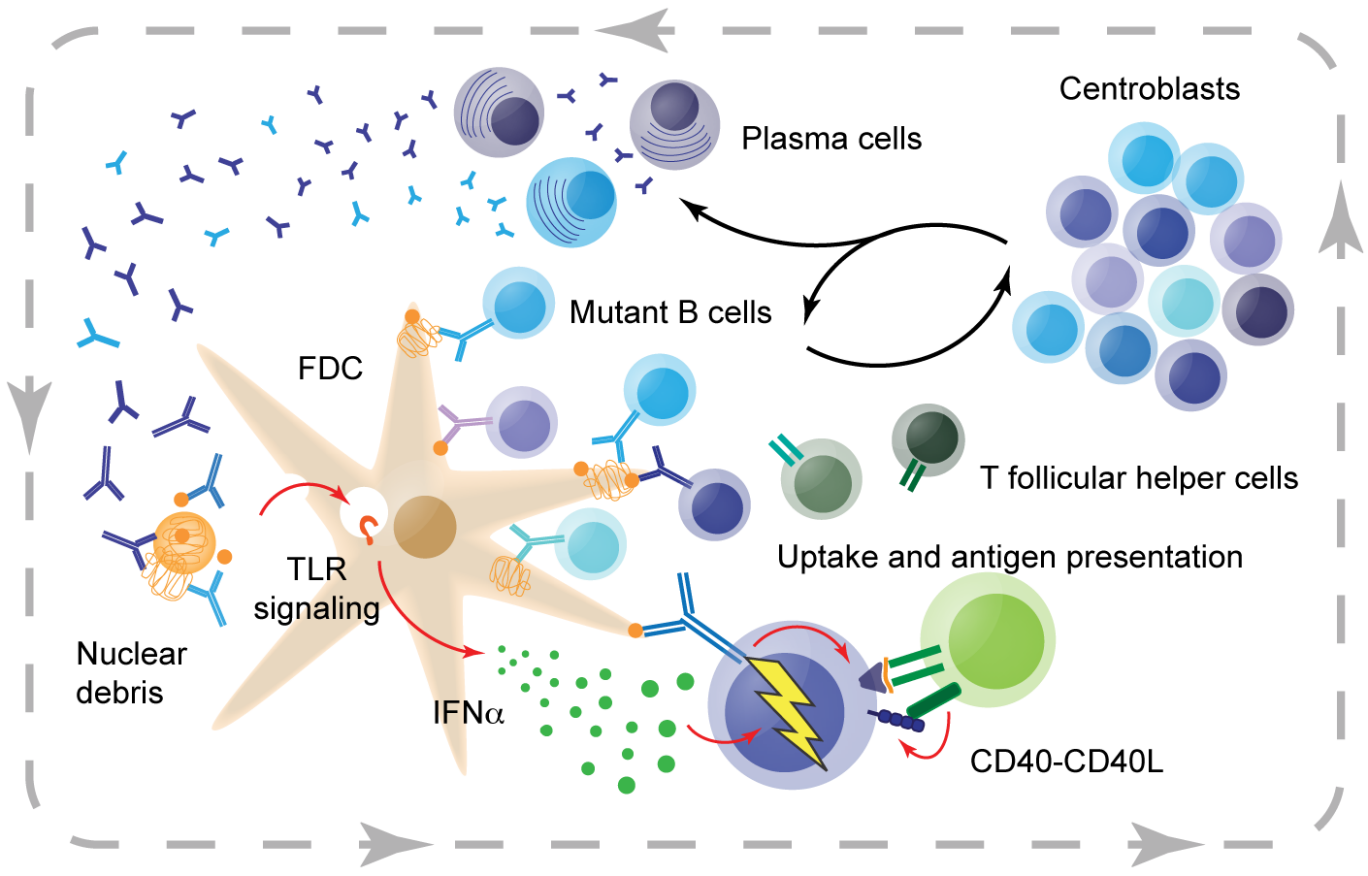
Model of the autoreactive germinal center and the main mechanisms fueling the 'furnace of autoreactivity' Follicular dendritic cells (FDCs) take up nucleolar debris, which in turn triggers endosomal TLR signaling, leading to interferon alpha production. Interferon alpha acts on protoautore active B cells, which upon antigen engagement take up nuclear components and present derived peptides to T follicular helper cells. T follicular helper cells stimulate the B cells through CD40L-CD40 interactions, among others, to enter or return to the dark zone of the germinal center. Resulting B cell centroblasts divide and hypermutate, then return to the light zone to probe FDCs for antigen, and the process is repeated. Autoantibodies produced by plasma cells derived from B cells in the germinal center facilitate immune complex loading, completing the vicious cycle of autoreactivity. Red arrows indicate the main driving signals and suggest possible points of intervention.
